# A Few Remarks on Phthisis, and on Its Physiological Treatment

**Published:** 1847-07

**Authors:** 


*A few Remarks on Phthisis, and on its Physiological Treatment.* By M. Bouchardat. Translated for the Medical Times by Alfred Markwick, Esq., Surgeon to the Western German Dispensary, and formerly Externe to the Venereal Hospital, Paris, &c.—The anatomical doctrine which prevails at the present day has, in more than one instance, substituted facts for errors; it has, in more than one disease, replaced bad therapeutics by a rational and efficacious mode of treatment, and has more than once, by pointing out to us the fallacy of science being already perfected, contributed to delay the period of useful and fruitful research. In pulmonary phthisis, in particular, the true nature of the disease consists, in the opinion of all our anatomico-pathological writers, in the specific alteration of the lungs; and it may be observed from its commencement, and followed throughout all its stages. This opinion being adopted, the origin of the disease is considered to be a specific irritation of the organ; and upon this supposition, medical men direct their treatment against the local affection, and torment their patients by the local application of leeches, cauteries, and moxas!

This opinion on the nature of pulmonary consumption is one of the worst that can be formed; the reality is not sought for, but a vain fancy followed, and the disease itself allowed to increase daily in severity, and at length to become incurable, without any useful means being resorted to oppose it.

I am about to defend an opinion which is to lead to quite a different mode of treatment, but which will often present, in its application, numerous difficulties, and require special researches to be made.

According to a careful microscopical examination, and a complete chemical analysis, the results of which I intend to publish elsewhere, it appears that tubercule is formed by the reunion of *particula*

*globules* which have no existence in the animal economy in the healthy state. These particular globules become developed spontaneously in the bodies of animals that are placed under certain circumstances which will presently be more particularly alluded to. These globules unite and coalesce, and become destroyed by giving rise to secondary products, and to fresh organized globules; they invade every organ, but more particularly the more susceptible ones—those, in fact, of the most *delicate* structure, such, for instance, as the lungs.

If this opinion be admitted *à priori*, and its value is proved by subsequent experience, we are then naturally led to inquire after the cause or causes which place the animal economy in such a condition that the tubercular globule becomes spontaneously developed. If we are fortunate enough to discover the cause or causes, then all doubts as to the treatment ceases; it becomes then truly physiological.

The majority of those physicians who at the present day adopt an opinion analogous to the one I have just expressed on the nature of the tubercular affection will reply without hesitation. But the true cause of the disease is a particular *diathesis;* and to them, this word diathesis is something mysterious—something inaccessible to our researches. This habit of being contented with words is but too common in pathology, and is almost as dangerous as being in error.

If we apply this method to the study of phthisis, we at once find that the tubercular globule becomes developed in individuals that are reduced either by bad nourishment, or by the excessive, continued, and unrepaired loss of some fluid essential to the animal economy, such as the lymph and serum, from too abundant suppurations, or the spermatic fluid from venereal excesses, &c.

It will be perceived that the causes of debility which may bring about the spontaneous evolution of the tubercular globule are both complex and numerous. I shall not now attempt to touch upon all parts of this problem, which, indeed, is beyond my capabilities; but shall merely take a case that has fallen under my own observation, and follow, with regard to it, the plan I have described; the case in question is one of tubercular affection occurring in a diabetic patient.

### *On the connection which exists between Diabetes and the Tubercular Disease.*

In all my writings on diabetes, I have alluded to the tubercular complication which has been very justly insisted on by several authors. In all the diabetic patients comprised in the first series of cases, on whom a *post-mortem* examination was made, and who did not die from any intercurrent accident, tubercles were found in the lungs.

I am convinced that many tubercular affections have originated in slight attact of diabetes which has been overlooked, and I cannot too strongly recommend medical men to carefully examine the urine of patients threatened with phthisis. By adhering to the rules I laid down when speaking of "the means of detecting the presence of starch sugar in urine, and of measuring its quantity;" it is possible in many cases to ward off or arrest an affection which is so difficult to be overcome when once it has manifested itself. I will not quit this interesting subject, of the affinity between diabetes and the tubercular disease, without making a few remarks, the truth of which must eventually be proved in practice.

In the majority of cases, the truly positive ætiology of pulmonary phthisis is still enveloped in great obscurity. Of late years some very exact researches have been made which have been the means of proving, with great certainty, the existence of tubercles when they are present in the lungs. The Parisian school has brought the methods of percussion and auscultation to the utmost perfection. One may almost foretel the alterations that will be found on *post-mortem* examination. This part of the science, the results of which act on the minds of students, has been cultivated with so much spirit that *physiological ætiology*, which alone can lead to a rational and really effectual mode of treatment, has, in consequence, been perhaps a little too much neglected. It is, no doubt, useful to be able to detect, either by the aid of instruments or by our own improved senses, those lesions which would escape a less practised ear; still it is of far greater importance to prevent them, and to stop, if possible, their progress.

The excellent understanding of a great number of celebrated physicians of our time has led them to abandon the uncertain doctrines of our predecessors, and to introduce into medicine the positive methods of the other sciences of observation. Great perseverance has been employed, not only to determine the physical alterations of the diseased organs or tissues, but also to find out the nature of these hidden alterations by means of a strict diagnosis; but, being unable to introduce into the research after the *true* causes this precision, the study of the nature of diseases has been repeatedly declared of but little importance, and those investigations which, in my opinion, can alone lead to a satisfactory mode of treatment, have been neglected. I am aware that these investigations are difficult, and that more than one false step has been made in this direction; but within the last fifty years sufficient progress has been made in both physics and chemistry to enable us to foresee the time when we shall be able to unravel the mysteries of many of the organic phenomena which have hitherto been considered as unfathomable. Medicine will never assume a truly scientific and exact character until, aided by physics and chemistry, it possesses positive data respecting the nature of diseases.

We will now attempt to handle the very difficult question of the physiological ætiology of the tubercular affection, and will proceed, as in the exact sciences, from the known to the unknown. We commence with the following principle, which may be considered as a law of pathology, viz:—

"When a patient, labouring under diabetes dies slowly from the constant progress of the disease without the occurrence of any other accident, tubercles are invariably found in the lungs after death."

Here, then, are individuals with sound lungs, in whom the development of tubercles may be foretold. Therefore, as we now know the nature of diabetes, we may, for this particular case, arrive at a positive ætiology of the tubercular affection.

In what does a diabetic patient chiefly differ from a man in health? More particularly in this, that in the healthy person, the feculent food dissolved in the digestive apparatus, and carried slowly into the circulation, is there completely consumed, no trace of it being afterwards found either in the fæces or in the urine ; whereas, in the diabetic, the feculents, rapidly converted into glucose in the stomach, are immediately absorbed ; and this glucose, being in too large a proportion in the circulating apparatus to be normally destroyed, becomes eliminated by the kidneys. Hence there are three important circumstances which distinguish the diabetic from the healthy individual, viz.:—1, the perversion of the functions of the stomach, causing a rapid solution of the feculents ; 2, the existence of a large quantity of glucose in the blood ; 3, great activity of the secreting organs of the urine to get rid of the glucose. Important consequences proceed from these differences.

The active powers of the digestive organs, and the secreting apparatus of the urine, are uselessly employed for the support and repair of the animal economy. The nature of the transformations which the nutritive fluid is continually undergoing is modified by the presence of a considerable proportion of glucose in the blood. The food dissolved in the digestive apparatus being no longer usefully employed, the patient is supported at the expense of himself; hence the emaciation and wasting, with all its results. Now, the necessary effect of this anomalous condition is the spontaneous production, and the localization in the lungs, of tubercular globules, which eventually, by their successive agglomeration, invade this organ and prevent its important functions.

The causes of the spontaneous evolution of tubercles for this particular condition are thus clearly established :—

1. Perversion in the digestion of feculents.
2. Presence in the blood of a variable proportion of glucose.
3. Elimination of the glucose by the kidneys.
4. Replacing the glucose eliminated, by the slow destruction of the fundamental principles of the blood, the muscles, and the other organs.

May not analogous circumstances be met with in the different conditions under which tubercles become developed in the lungs or in other organs?

Apart from these cases, in which tubercles are developed as the result of the perversion in the digestion of feculents, can we not easily understand that other perversions in the important function of nutrition may cause the development of the tubercular affection ? I hope, when our great work on digestion is completed, to be able to return to the nature of these perversions, which may be suspected after what we have published. I think, therefore, it would be of the greatest importance to make some careful and accurate investigations into the manner in which the digestion and the assimilation of the different aliments takes place in persons in whom phthisis has just commenced or who are threatened with this disease. We should then establish an equation, as I have done with respect to diabetes; the food and drink would form its first term, and the principles contained in the fæces, the urine, and the other products of secretion or otherwise, the nature and quantity of which could be appreciated, the second. These investigations would lead to results equally precise with those I have obtained in diabetes. We may resume the contents of this paper in the following propositions:—

1. The cause of the development of tubercles in the lungs of diabetes is a defect in nutrition and assimilation.

2. The tubercular affection has its origin, much more frequently than is imagined, in a defect in nutrition and assimilation, which cannot be known, and which can only be remedied by establishing an exact balance between the *ingesta* and the *excreta*.

3. It likewise originates in the excessive, continued, and unrestrained losses of fluids that are essential to the economy.

4. When a patient becomes emaciated it is important to ascertain as soon as possible the cause of the emaciation, and to remedy it; we should thus alter the conditions which give rise to the spontaneous evolution of the tubercular globules.—*Lond. Med. Times.*

